# Supplementary material for: Population, distribution, biomass, and economic value of Equids in Ethiopia
Source: PLoS One. 2024 Mar 22;19(3):e0295388. doi: 10.1371/journal.pone.0295388 (PMC10959329; doi:10.1371/journal.pone.0295388)
Supplement: S1 File — (DOCX) [file pone.0295388.s001.docx]

**S1 File.** Interview guide for current market price, number of service day and rental value.

**Interview guide**

The Brooke and the University of Liverpool have partnered for a four-year project to understand how to address impact of animal diseases effectively and efficiently through systematic data collection and analysis in order to quantify and understand the burden of animal diseases on working equids. Girma Asteraye (A PhD student) is representing the Brooke in Ethiopia and PhD work fully funded by the Brooke. The outcome from the research is vital to Ethiopia and globally by producing a standardized, holistic approach to assessment of the burden of animal diseases in working equids.

Please use your intern staff and team to interview and fill-in the attached table that requests three questions,

1. Current market price of equids
2. The number of service days worked by equids,
3. Daily average income from equids

***** Please ensure that oral consent is acquired before beginning the interview.

**Current market price of equids as of November 2021**

| **Market place/ district** | **Donkey** | | **Horse** | | **Mule** | |
| --- | --- | --- | --- | --- | --- | --- |
|  | **Young** | **Adult** | **Young** | **Adult** | **Young** | **Adult** |
|  |  |  |  |  |  |  |
|  |  |  |  |  |  |  |

**Note:** Young [age below 3 years] and Adult [ age 3 years and above]

**Number of service days worked by equids.**

| Name of the district | **Service day per a week** | | | | | | | | |
| --- | --- | --- | --- | --- | --- | --- | --- | --- | --- |
|  | **Donkey** | | | **Horse** | | | **Mule** | | |
|  | **Min** | **Max** | **Average** | **Min** | **Max** | **Average** | **Min** | **Max** | **Average** |
|  |  |  |  |  |  |  |  |  |  |

**Note:** number of service days does not consider working hours

**Daily average income from equids as of November 2021**

| **District** | **Donkey cart** | **Garry Horse** | **Cart horse** | **Cart Mule** | **Remark** |
| --- | --- | --- | --- | --- | --- |
|  |  |  |  |  |  |

**Who to Contact:**

You are welcomed to reach out the research team if you and interviewee are worried for any reason or questions regarding the study.

To make queries later, please contact:

• Girma Birhan Asteraye ([Girma.Asteraye@liverpool.ac.uk](mailto:Girma.Asteraye@liverpool.ac.uk) phone : +251918034060), or

• Prof. Jonathan Rushton ([jrushton@liverpool.ac.uk](mailto:jrushton@liverpool.ac.uk) ).
