# Supplementary material for: Population, distribution, biomass, and economic value of Equids in Ethiopia
Source: PLoS One. 2024 Mar 22;19(3):e0295388. doi: 10.1371/journal.pone.0295388 (PMC10959329; doi:10.1371/journal.pone.0295388)
Supplement: S1 Fig — (DOCX) [file pone.0295388.s003.docx]

S2 Fig: The proportion of equids biomass(a) and stock monetary value(b) with total livestock biomass and stock economic value (2020/21 CSA population & MoTI market price data).
