# Supplementary material for: Population, distribution, biomass, and economic value of Equids in Ethiopia
Source: PLoS One. 2024 Mar 22;19(3):e0295388. doi: 10.1371/journal.pone.0295388 (PMC10959329; doi:10.1371/journal.pone.0295388)
Supplement: Attachment — Submitted filename: Response to reviewers_2.docx [file pone.0295388.s005.docx]

**Response to reviewer -1**

I would like to thank the reviewers and the editor once more for their insightful comments and questions. We attempted to incorporate feedback and give clarification responses, written in purple.

Thank you very much!

**Response to reviewer #1:**

Reviewer #1: Thank you for providing this improved manuscript. Please note that it would be helpful to provide a version with marked up tracked changes that show where changes have been made to the document, not wholesale deletions and reinsertions. Additionally it would be helpful if the responses consistently contained line numbers that refer to the most recent version of the manuscript. It is very time consuming to re-review a document presented in this way. The document I have access to has four different versions of the paper -I have based this review on the one starting on page 68. This means I may miss some changes, as I cannot reference them by line number, or comment on parts of the manuscript that are unchanged.

Dear reviewer-1: Please accept my apologies for making the review challenging; I will work properly after this. And apologise for not returning my response within fortnights, I was out of the city for data collection, and the internet connectivity was very limited, which made it difficult for me to send my response in time. I appreciate your patience and understanding in this regard. Thank you.

Please refer to the indicated line numbers in the recent version of the manuscript

Generally, two main comments still require some changes. It is good to see that the suggestion about including a numerical range to represent the sampling uncertainty. But,

- What does the ranges presented represent and how were they calculated? – confidence intervals? Standard deviation? These should inform your sensitivity analysis, which in insufficiently described in the methods, results and discussion.
  Okay, the range shown is a 95% confidence interval (CI); because the CSA estimates livestock population and indicates a standard error, we calculated the confidence interval to take into account the uncertainty associated with sampling variability; we have calculated using the formula

Confidence Interval = The equids population mean ± (Z * Standard Error)

Mean: The equids population mean

Z: ≈ 1.96 as CSA calculated the CI at 95%

Standard Error: the SE value from CSA

The sampling uncertainty was expressed by calculating a confidence interval from CSA livestock population estimates and standard errors. This is now included in the method section line 173-74.

**Some additional points**
Line 68: In this case “demonstrated that equids provide 14% of annual household income … which was comparable to other livestock species in that study”.

Yes, as per the result of Admassu and Shiferaw (2011), working equids contribute 14% of household income and which was comparable to other livestock species. I accept the comment - line 69

Line 77: This still feels like a contradiction to me and would benefit from some critical thinking (with reference to line 90-91 as well)
I have incorporated necessary change for the sentence as per the comment received - line 81-82

Line 81: The manuscript does not reflect the response to reviewers

I have made changes to the paragraph ( line 79 to 83) based on the feedback I received.

Line 173: suggest: “The equid population percentage change was calculated by dividing the difference between the initial equine population in 2004 and the population in 2020 by the initial population and multiplying by 100”
Thank you and I accept your suggestion fully, and addressed, accordingly, please see line (170-72)

Table 2: explain what these numbers are a percentage of – all equid holders or all livestock holders. Make sure that the title represents the tables contents – it does not show the change, simply the populations in the two years

This proportion was determined based on total livestock holdings, and I have updated the title to reflect this (line 203-4).

Table 4 appears to repeat table 3 (although the numbers are slightly different). Can you include a clear explanation?

As mentioned in the methodology section, the CSA separated the equine population based on species, sex, and purpose. This information was used to calculate the biomass and the monetary value of the stock. The slight difference in the number between the total (Table 3) and the categorized (Table 4) is a result of the disaggregation process. I have included the explanation for the difference, please see line 237-8.

Line 212: “Because the data is insufficient, we did not estimate the effective service days of
equids for draught usage.” – but then this seems to have been done in table 5

As it indicated in method section (line 140-42) rental value and number of service days for draft usage, were obtained from a study done by Metaferia et al (2010) for calculation to estimates of service value of equids. Therefore, the draught usage service days indicated in table 5 are from Metaferia et al (2010). Kindly, If I am expected to write under a table 5 as a note, I can do that.

Line 269: Which scientific growth rate estimates are you referring to?

I should not have used the word "scientific" in that sentence. I was referring to the unexpected growth of the equid population in 2006 with 19.2%, which is quite different from the rest of the consecutive years, and if the equid birth rate is one foal per year (which is equivalent to a 100% foaling rate), this would not result in the type of growth CSA indicated in 2006. Please see (lines 274 and 75) for a correction.

Line 315 onwards: I think it would still be beneficial to discuss these other estimates in more depth, even if they do not have clearly defined methodologies (even including in the manuscript that the methodologies are not clearly defined.)
I have tried to include their methods and discuss with our result, can you please see line 321-330

The manuscript would benefit from a thorough proofread: e.g. consistency of decimal places, some verb agreements, use of data as either a singular or plural throughout, not a mix

I attempted to make decimal places consistent manly in the tables by approximating.

Thank you very much and this will definitely strengthen our paper.

Respectfully, Girma (corresponding author)
